# Predictive factors for tooth loss during supportive periodontal therapy in patients with severe periodontitis: a Japanese multicenter study

**DOI:** 10.1186/s12903-019-0712-x

**Published:** 2019-01-15

**Authors:** Takahisa Hirata, Shinya Fuchida, Tatsuo Yamamoto, Chieko Kudo, Masato Minabe

**Affiliations:** 1 Division of Periodontology, Department of Oral Interdisciplinary Medicine, Graduate School of Dentistry, Kanagawa Dental University, Yokosuka, Japan; 2 Division of Dental Sociology, Department of Oral Science, Graduate School of Dentistry, Kanagawa Dental University, Yokosuka, Japan; 3 Institute of Medical Corporation Shinsekai, Tokyo, Japan

**Keywords:** Supportive periodontal therapy, Periodontal disease, Tooth loss, Periodontal risk assessment, Therapy-resistant periodontitis

## Abstract

**Background:**

Supportive periodontal therapy (SPT) must take individual patient risk factors into account. We conducted a multicenter joint retrospective cohort study to investigate the value of modified periodontal risk assessment (MPRA) and therapy-resistant periodontitis (TRP) assessment as predictive factors for tooth loss due to periodontal disease in patients with severe periodontitis during SPT.

**Methods:**

The subjects were 82 patients from 11 dental institutions who were diagnosed with severe periodontitis and continued SPT for at least 1 year (mean follow-up = 4.9 years) between 1981 and 2008. The outcome was tooth loss due to periodontal disease during SPT. The Cox proportional hazards model was used to analyze sex, age, diabetes status, smoking history, number of periodontal pockets measuring ≥6 mm, rate of bleeding on probing, bone loss/age ratio, number of teeth lost, MPRA, and TRP assessment as explanatory variables.

**Results:**

Univariate analysis showed that loss of ≥8 teeth by the start of SPT [hazard ratio (HR) 2.86], MPRA score indicating moderate risk (HR 8.73) or high risk (HR 11.04), and TRP assessment as poor responsiveness to treatment (HR 2.79) were significantly associated with tooth loss (*p* < 0.05). In a model in which the explanatory variables of an association that was statistically significant were added simultaneously, the HR for poor responsiveness to treatment and ≥8 teeth lost was significant at 20.17 compared with patients whose TRP assessment indicated that they responded favorably to treatment and who had lost <8 teeth by the start of SPT.

**Conclusion:**

MPRA and TRP assessment may be useful predictive factors for tooth loss due to periodontal disease during SPT in Japanese patients with severe periodontitis. Additionally, considering the number of teeth lost by the start of SPT in TRP assessment may improve its predictive accuracy.

## Background

Prevention and control of periodontal disease are an important issue for improving dental and oral health. A large-scale Japanese survey in 2005 found that periodontal disease was the major cause of permanent tooth extraction and that it accounted for a particularly high rate of extractions among those aged 30–60 years [1]. The results of Survey of Dental Diseases of 2016 showed that although the number of people with ≥20 teeth had increased in all age groups, the proportion of those with periodontal pockets measuring ≥4 mm was high in almost all age groups and was particularly high among older people [2].

Supportive periodontal therapy (SPT) is treatment intended to stabilize the long-term condition of periodontal tissue after basic periodontal treatment or periodontal surgical treatment [3], which has been demonstrated to be effective [4, 5]. Risk factors for individual patients must be taken into consideration in order to carry out SPT effectively, and previous studies have addressed risk factors for the recurrence of periodontitis and for tooth loss. Risk factors at the tooth level include the presence of probing depth (PD) ≥6 mm [6] and the type of tooth [7], and those at the patient level include age [8], genetic polymorphism [8], smoking history [8, 9], and systemic conditions such as diabetes [7]. Assessing these risk factors in combination rather than separately improves the accuracy with which tooth loss due to the recurrence of periodontitis can be predicted [10–12]. Additionally, several different models have been proposed as risk assessment indices, including the Periodontal Risk Calculator [10] and periodontal risk assessment (PRA) [11, 12].

Among these models, PRA takes account not only of the condition of periodontal tissue in terms of factors such as deep PD and percentage of sites positive for bleeding on probing (BOP), but also of smoking history and systemic conditions, and is widely used as a comprehensive risk assessment index at the patient level [13]. Previous studies of the association between PRA and prognosis during SPT for patients with moderate or severe periodontitis have found that patients assessed as high-risk by PRA are significantly more likely to experience tooth loss than those assessed as low-risk [8, 14, 15]. Recent studies have also found that the accuracy of prediction can be improved by excluding genetic risk factors (interleukin-1 genotype positive) from PRA [16] and changing the bone loss calculation method for determining the bone loss/age ratio [17]. Additionally, there is a need both to verify the value of combination risk assessment indices and improve the accuracy of prognosis predictions by focusing on individual risk factors. However, most previous studies of the association between PRA and tooth loss in patients with periodontitis have been carried out in Europe [18]. Moreover, it is unclear whether these associations apply in Japanese due to racial differences and differences in prevalence [19]. Risk assessment must also be universally applicable in dental institutions providing care in a variety of different formats. However, to date, few multicenter studies have been carried out.

In Japan, the therapy-resistant periodontitis (TRP) assessment diagnostic tool has been shown to be useful as a predictive factor for tooth loss during SPT [20]. TRP assesses the improvement rate of deep periodontal pockets in basic periodontal treatment based on previous studies [21, 22]. TRP is defined as <70% of sites with PD ≥6 mm at initial examination that improved by ≥2 mm after basic periodontal treatment. TRP diagnosis was found to be a significant risk factor for tooth loss with an odds ratio of 2.81 (*p* = 0.006) [20]. However, the outcome of tooth loss includes teeth lost to extractions due to causes other than periodontal disease. Furthermore, as the effect of differences between patients in the duration of SPT was also not taken into account in that analysis, further studies are required.

In this study, we carried out a multicenter joint retrospective cohort study to investigate the value of modified periodontal risk assessment (MPRA) and TRP assessment as predictive factors for tooth loss due to periodontal disease during SPT in patients with severe periodontitis. For MPRA, we modified the cut-off value of counting sites of PD from ≥5 mm to ≥6 mm [17] because subjects were restricted to those having severe periodontitis. Additionally, we used a Cox proportional hazards model that took follow-up period into account. We also evaluated the applicability of individual risk factors with the aim of further improving the accuracy of prediction.

## Methods

### Patients

**Fig. 1:**
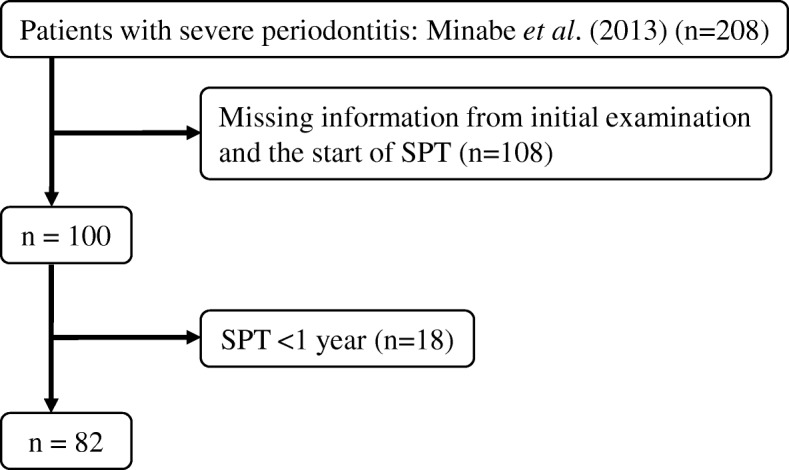
Flow chart of the subjects for analysis

To minimize variation among institutions in treatment strategies and treatments during SPT, the participating institutions comprised a university hospital and 10 dental clinics between 1981 and 2008. In the institutions, specialist periodontists and dental hygienists board-certified by the Japanese Society of Periodontology were employed full-time. As the subjects of a previous study [20], out of 1,614 patients with periodontitis undergoing regular examinations in these 11 institutions who had moved on to SPT after re-evaluation following the completion of basic periodontitis treatment or periodontal surgical treatment, we initially selected 208 patients with severe periodontitis on the basis of the following criteria: age ≥20 years at initial examination, ≥16 remaining teeth, and ≥1 periodontal pocket measuring ≥6 mm. In this study, severe periodontitis was defined as ≥1 periodontal pocket measuring ≥6 mm. Of these patients, 108 patients were excluded because of missing patient information from initial examination and the start of SPT, and 18 patients were excluded because they underwent SPT for <1 year, leaving 82 patients (34 men and 48 women) as the subjects of our analysis (Fig. 1). For 79 of the 108 patients who were excluded, either there was no X-ray image at the start of SPT to use for calculating the bone loss/age ratio or the X-ray image was unclear.

The study protocol was approved by the Ethics Committee of Aichi Gakuin University School of Dentistry (no. 276) and the Research Ethics Committee of Kanagawa Dental University (no. 388).

### Risk assessment indices

Data on patient characteristics at the time of initial examination and the start of SPT were collected from medical records. Data comprised sex, age, diabetes status (under treatment), smoking history, number of sites with PD ≥6 mm (6 points measured per tooth), percentage of sites positive for BOP, bone loss/age ratio (percentage of bone loss at the site of greatest tooth loss in the alveolar ridge on the surface adjacent to the molar region measured with the Schei ruler [23], divided by age), and the number of teeth lost out of a maximum of 28 (excluding the third molars).

MPRA was used as an overall risk assessment index and was scored out of a total of 6 risk factors. The patients were classed into three groups: patients with ≥5 low-risk factors at the start of SPT were classed as low risk; patients with ≥2 moderate risk factors (including patients with 1 moderate and 1 high-risk factor) were classed as moderate risk; and patients with ≥2 high-risk factors were classed as high risk. However, because smoking history is evaluated solely on the basis of experience, it was used as a moderate risk factor.

We also used the TRP assessment [20], which calculates the proportion of sites of PD ≥6 mm at initial examination that improved by ≥2 mm after basic periodontal treatment. Patients in whom improvement was evident at ≥70% of sites were classed as favorable responsiveness to treatment, and those in whom it was evident in <70% as poor responsiveness.

### Statistical analysis

After descriptive analysis, an investigation was carried out using the Cox proportional hazards model with tooth loss due to periodontal disease during SPT as the outcome. Patient characteristics and risk evaluation indices at the time of initial examination and the start of SPT were included as explanatory variables. Specifically, for each explanatory variable, univariate analysis was performed and the hazard ratio (HR) and 95% confidence interval (95% CI) were calculated. For patients who underwent tooth extraction due to periodontal disease during SPT the follow-up period was considered to be from the date of the first appointment during SPT until the date of the extraction. Additionally, for those who did not undergo extraction it was considered to be from the date of the first appointment during SPT to the date of the last (most recent) appointment, with the former regarded as the occurrence of an event and the latter as terminated (follow-up completed).

Multivariate analysis simultaneously adding all explanatory variables for which a significant association was evident in univariate analysis was then performed, and its HR and 95% CI were calculated.

IBM SPSS Statistics 23 (IBM Japan, Ltd., Tokyo, Japan) was used for statistical analysis, with *p* < 0.05 regarded as significant.

## Results

Table 1 shows the characteristics of the subjects included in our analysis at the time of initial examination and at the start of SPT. At the time of initial examination, the mean age was 47.2 years, the mean number of sites with PD ≥6 mm was 39.9, the mean percentage of sites positive for BOP was 50.6%, the mean bone loss/age ratio was 1.51, and the mean number of teeth lost was 2.6/28. The mean number of teeth extracted between basic periodontal treatment and the start of SPT was 1.4, and the mean number of teeth lost at the start of SPT was 4.0/28. The mean follow-up period after the start of SPT was 4.9 years, and the total follow-up period for all 82 patients was 402.9 person-years, with 16 patients (19.5%) losing teeth.

**Table 1:** Patient characteristics

		n (%)	
		Initial examination	Start of SPT
Sex	Male	34 (41.5)	
	Female	48 (58.5)	
Age	≤ 34	9 (11.0)	5 (6.1)
	35–44	25 (30.5)	20 (24.4)
	45–54	27 (32.9)	27 (32.9)
	55–64	16 (19.5)	24 (29.3)
	≥ 65	5 (6.1)	6 (7.3)
Diabetes status	Non-DM	80 (97.6)	80 (97.6)
	DM	2 (2.4)	2 (2.4)
Smoking history	Non-smoker	62 (75.6)	62 (75.6)
	Smoker	20 (24.4)	20 (24.4)
Number of sites with PD ≥ 6 mm	< 8	6 (7.3)	80 (97.6)
	≥ 8	76 (92.7)	2 (2.4)
BOP	< 25%	13 (15.9)	69 (84.1)
	≥ 25%	69 (84.1)	13 (15.9)
Bone loss/age ratio	< 1.0	16 (19.5)	25 (30.5)
	≥ 1.0	66 (80.5)	57 (69.5)
Number of teeth lost	< 8	74 (90.2)	66 (80.5)
	≥ 8	8 (9.8)	16 (19.5)
MPRA	Low risk		27 (32.9)
	Moderate risk		34 (41.5)
	High risk		21 (25.6)
TRP assessment	Favorable		61 (74.4)
	Poor		21 (25.6)

*DM* diabetes mellitus, *PD* probing depth, *BOP* bleeding of probing, *MPRA* modified periodontal risk assessment, *TRP* therapy-resistant periodontitis

An investigation of the possible association between tooth loss due to periodontal disease during SPT and patient characteristics and risk assessment indices at the time of initial examination did not identify any significant associations (Table 2).

**Table 2:** Association between the number of teeth lost due to periodontal disease during SPT and periodontal risk factors at the time of initial examination (Cox proportional hazards model)

Initial examination	Total	Teeth loss due to periodontal disease during SPT			HR	95% CI	*p* value
Variables		n	Person-years	Rate			
Sex							
Male	34	7	159.3	0.0440	1.00		
Female	48	9	243.7	0.0369	0.77	0.29–2.08	0.610
Age							
≤ 34	9	3	31.8	0.0945	1.00		
35–44	25	4	153.6	0.0260	0.28	0.61–1.26	0.096
45–54	27	7	105.2	0.0666	0.76	0.19–2.97	0.689
55–64	16	1	91.3	0.0110	0.11	0.01–1.10	0.060
≥ 65	5	1	21.2	0.0472	0.51	0.05–5.00	0.562
Diabetes							
Non-DM	80	16	398.1	0.0402	1.00		
DM	2	0	4.8	0.0000	0.05	0.00–6.27 × 10^7^	0.777
Smoking history							
Non-smoker	62	11	301.2	0.0365	1.00		
Smoker	20	5	101.8	0.0491	1.41	0.49–4.09	0.522
Number of sites with PD ≥ 6 mm							
< 8	6	2	60.6	0.0330	1.00		
≥ 8	76	14	342.3	0.0409	1.39	0.30–6.51	0.680
BOP							
< 25%	13	5	87.5	0.0571	1.00		
≥ 25%	69	11	315.4	0.0349	0.67	0.22–1.98	0.465
Bone loss/age ratio							
< 1.0	16	1	67.8	0.0147	1.00		
≥ 1.0	66	15	335.1	0.0448	3.19	0.42–24.17	0.262
Number of teeth lost							
< 8	74	14	374.3	0.0374	1.00		
≥ 8	8	2	28.6	0.0700	2.27	0.48–10.71	0.300

*HR* hazard ratio, *CI* confidence interval, *DM* diabetes mellitus, *PD* probing depth, *BOP* bleeding of probing

Table 3 shows the results of an investigation of the association between tooth loss due to periodontal disease during SPT and patient characteristics and risk assessment indices at the start of SPT. There were significant associations with the loss of ≥8 teeth by the start of SPT (versus patients who had lost <8 teeth: HR 2.86, 95% CI 1.02–8.01), MPRA score indicating moderate risk (versus low-risk patients: HR 8.73, 95% CI 1.10–69.09) or high risk (versus low-risk patients: HR 11.04, 95% CI 1.31–93.37), and TRP assessment as poor responsiveness to treatment (versus favorable responsiveness to treatment: HR 2.79, 95% CI 1.05–7.44) (*p* < 0.05).

**Table 3:** Association between the number of teeth lost due to periodontal disease during SPT and periodontal risk factors at the start of SPT (Cox proportional hazards model)

Start of SPT	Total	Teeth loss due to periodontal disease during SPT			HR	95% CI	*p* value
Variables		n	Person-years	Rate			
Age							
≤ 34	5	1	14.2	0.0706	1.00		
35–44	20	3	101.5	0.0296	0.36	0.04–3.57	0.380
45–54	27	9	138.0	0.0652	0.76	0.09–6.33	0.796
55–64	24	2	122.4	0.0163	0.20	0.02–2.30	0.198
≥ 65	6	1	26.8	0.0373	0.46	0.03–7.51	0.586
Diabetes							
Non-DM	80	16	398.1	0.0402	1.00		
DM	2	0	4.8	0.0000	0.05	0.00–6.27 × 10^7^	0.777
Smoking history							
Non-smoker	62	11	301.2	0.0365	1.00		
Smoker	20	5	101.8	0.0491	1.41	0.49–4.09	0.522
Number of sites with of PD ≥ 6 mm							
< 8	80	16	396.7	0.0403	1.00		
≥ 8	2	0	6.3	0.0000	0.05	0.00–3.81 × 10^6^	0.743
BOP							
< 25%	69	13	356.9	0.0364	1.00		
≥ 25%	13	3	46.0	0.0652	2.31	0.61–8.82	0.219
Bone loss/age ratio							
< 1.0	25	4	116.9	0.0342	1.00		
≥ 1.0	57	12	286.0	0.0420	1.21	0.39–3.76	0.742
Number of teeth lost							
< 8	66	10	335.1	0.0298	1.00		
≥ 8	16	6	67.8	0.0885	2.86	1.02–8.01	0.046
MPRA							
Low risk	27	1	153.7	0.0065	1.00		
Moderate risk	34	9	161.1	0.0559	8.73	1.10–69.09	0.040
High risk	21	6	88.2	0.0681	11.04	1.31–93.37	0.027
TRP assessment							
Favorable	61	8	296.6	0.0270	1.00		
Poor	21	8	106.3	0.0752	2.79	1.05–7.44	0.040

*HR* hazard ratio, *CI* confidence interval, *DM* diabetes mellitus, *PD* probing depth, *BOP* bleeding of probing, *MPRA* modified periodontal risk assessment, *TRP* therapy-resistant periodontitis

Table 4 shows models in which MPRA score, the number of teeth lost by the start of SPT (a component of MPRA), and TRP assessment, all of which exhibited a significant association in univariate analyses, were added simultaneously. In Model 1, in which MPRA score and TRP assessment were added simultaneously, TRP assessment was no longer significant, but MPRA score indicating high risk had a significantly higher HR compared with a low-risk score (HR 11.17, 95% CI 1.31–94.90). In Model 2, in which the number of teeth lost by the start of SPT and TRP assessment was added simultaneously, both variables were significant. A further investigation using a combination of two variables in Model 2 found that the HR for poor responsiveness to treatment and ≥8 teeth lost was 20.17 (95% CI 3.45–118.12) and was significantly high with respect to patients whose TRP assessment indicated that they were favorably responsive to treatment and who had lost <8 teeth by the start of SPT (Table 5).

**Table 4:** Associations between the number of teeth lost due to periodontal disease during SPT and risk assessment indices (Cox proportional hazards model)

	Model 1			Model 2		
	HR	95% CI	*p* value	HR	95% CI	*p* value
MPRA						
Low risk	1.00					
Moderate risk	7.76	0.98–61.56	0.053			
High risk	11.17	1.31–94.90	0.027			
Number of teeth lost						
< 8				1.00		
≥ 8				4.06	1.41–11.65	0.009
TRP assessment						
Favorable	1.00			1.00		
Poor	2.62	0.97–7.07	0.057	4.41	1.44–13.49	0.009

*HR* hazard ratio, *CI* confidence interval, *MPRA* modified periodontal risk assessment, *TRP* therapy-resistant periodontitis

**Table 5:** Associations between the number of teeth lost due to periodontal disease during SPT and a combination of TRP assessment and the number of teeth lost by the start of SPT (Cox proportional hazards model)

TRP assessment	Number of teeth lost	Total	Teeth loss due to periodontal disease during SPT			HR	95% CI	*p* value
			n	Person-years	Rate			
Favorable	< 8	47	4	234.9	0.0170	1.00		
Favorable	≥ 8	14	4	61.7	0.0649	3.70	0.91–14.98	0.067
Poor	< 8	19	6	100.2	0.0599	3.48	0.98–12.35	0.053
Poor	≥ 8	2	2	6.2	0.3243	20.17	3.45–118.12	0.001

*HR* hazard ratio, *CI* confidence interval, *TRP* therapy-resistant periodontitis

## Discussion

In this study, we investigated risk assessment indices that predicted tooth loss due to periodontal disease during SPT in patients diagnosed with severe periodontitis and undergoing treatment by specialist periodontists at 11 institutions in Japan. We found that PRA score, which is widely used in Europe, is valid in Japan. This is consistent with the results of studies in Germany [8, 16], France [24], Switzerland [14], Brazil [15], and the United Kingdom [25], demonstrating the validity of PRA as a comprehensive risk assessment index. In terms of the individual component factors of PRA, in this study, the number of teeth lost was significantly associated with the number of teeth lost during SPT, a result consistent with those of previous studies [15, 24]. With respect to the other component factors of PRA score, with the exception of the number of sites with PD ≥6 mm, some previous studies have found significant associations with other component factors, while others have found non-significant associations. No previous study, including this one, has identified a significant association with the presence of high frequencies of deep residual pockets [8, 14–16, 24, 25].

The TRP assessment, which is under consideration in Japan, switched the outcome from extractions for any reason to extractions due to periodontal disease in response to an issue raised in a previous study [20] and has also been found to be effective when the duration of SPT is taken into account. Our results suggested that its predictive accuracy could be further improved by taking account of the number of teeth lost by the start of SPT. Patients who have lost more teeth may be at higher risk of tissue breakdown due to periodontal disease, which may cause further tooth loss in the future. Previous studies have also shown that the greater the number of teeth lost, the higher the risk for further tooth loss during SPT or the maintenance phase [15, 26], and the use of a combination of PRA and TRP assessment may further improve predictive accuracy.

The patients who were the subjects of our analysis were similar to those of previous studies of the effectiveness of PRA in terms of age and severity of periodontitis [8, 14–16, 24, 25]. However, the number of study subjects and the mean number of teeth lost during SPT and the overall follow-up period were both somewhat smaller. A comparison of previous studies that, like this study, also included patients with both aggressive and chronic periodontitis [8, 24] showed that the mean number of teeth lost was smaller in this study. This suggested that in this study SPT contributed to preventing tooth loss due to periodontal disease, further reinforcing the importance of SPT after periodontal treatment.

With respect to the study design, most previous studies have used a logistic regression model. However, although some studies used a uniform duration of SPT [15] or a model that took the duration of SPT into account [14], a couple failed to consider differences in the duration of SPT [8, 24]. Although some studies took tooth loss due to periodontal disease as the outcome [14, 24], most used tooth loss for any reason [8, 15, 16, 25]. On this point, in our study, we used a Cox proportional hazards model that took differences in the duration of SPT into account and restricted tooth loss to that due to periodontal disease, enabling the risk of periodontal disease to be more accurately predicted.

Finally, this study has some advantages. This was a multicenter joint study performed in both a university hospital and dental clinics, and the consistency of periodontal treatment was assured because diagnosis and treatment were performed by specialist periodontists. This study also used a Cox proportional hazards model limited to extractions due to periodontal disease. However, it had the limitations that many subjects were excluded because of difficulties in calculating the bone loss/age ratio, it included only two patients with diabetes, smoking history was evaluated solely on the basis of experience rather than quantitatively, and the bone loss/age ratio was overestimated in younger patients with major bone loss [17]. Furthermore, we did not evaluate bifurcation lesions, which have been shown to be associated with tooth loss in previous studies [27–29]. Moreover, modification of cut-off values of PD from ≥5 mm to ≥6 mm might result in underestimation of the predictability of the PRA model. Because only patients with severe periodontitis were included and residual sites with PD ≥6 mm are known as incompletely treated sites [6], we modified the model. Further studies of more patients will be required to confirm our results.

## Conclusion

PRA and TRP assessment may be useful predictive factors for tooth loss due to periodontal disease during SPT in Japanese patients with severe periodontitis. Additionally, considering the number of teeth lost by the start of SPT in TRP assessment may improve its predictive accuracy.

## Abbreviations

BOP: Bleeding on probing
CI: Confidence interval
HR: Hazard ratio
MPRA: Modified periodontal risk assessment
PD: Probing depth
PRA: Periodontal risk assessment
SPT: Supportive periodontal therapy
TRP: Therapy-resistant periodontitis
